# Dissolved organic carbon in streams within a subarctic catchment analysed using a GIS/remote sensing approach

**DOI:** 10.1371/journal.pone.0199608

**Published:** 2018-07-06

**Authors:** Pearl Mzobe, Martin Berggren, Petter Pilesjö, Erik Lundin, David Olefeldt, Nigel T. Roulet, Andreas Persson

**Affiliations:** 1 Department of Physical Geography and Ecosystem Science, Lund University, Lund, Skåne, Sweden; 2 GIS Centre, Lund University, Lund, Skåne, Sweden; 3 Climate Impacts Research Centre, Department of Ecology and Environmental Science, Umeå University, Umeå, Sweden; 4 Agricultural, Life and Environmental Sciences: Renewable Resources, University of Alberta, Edmonton, Alberta, Canada; 5 Department of Geography, McGill University, Montreal, Quebec, Canada; Michigan State University, UNITED STATES

## Abstract

Climate change projections show that temperature and precipitation increases can alter the exchange of greenhouse gases between the atmosphere and high latitude landscapes, including their freshwaters. Dissolved organic carbon (DOC) plays an important role in greenhouse gas emissions, but the impact of catchment productivity on DOC release to subarctic waters remains poorly known, especially at regional scales. We test the hypothesis that increased terrestrial productivity, as indicated by the normalized difference vegetation index (NDVI), generates higher stream DOC concentrations in the Stordalen catchment in subarctic Sweden. Furthermore, we aimed to determine the degree to which other generic catchment properties (elevation, slope) explain DOC concentration, and whether or not land cover variables representing the local vegetation type (e.g., mire, forest) need to be included to obtain adequate predictive models for DOC delivered into rivers. We show that the land cover type, especially the proportion of mire, played a dominant role in the catchment’s release of DOC, while NDVI, slope, and elevation were supporting predictor variables. The NDVI as a single predictor showed weak and inconsistent relationships to DOC concentrations in recipient waters, yet NDVI was a significant positive regulator of DOC in multiple regression models that included land cover variables. Our study illustrates that vegetation type exerts primary control in DOC regulation in Stordalen, while productivity (NDVI) is of secondary importance. Thus, predictive multiple linear regression models for DOC can be utilized combining these different types of explanatory variables.

## 1. Introduction

In subarctic and arctic regions, much of the carbon is in the form of soil organic carbon (SOC), in soil that is partly frozen (permafrost) [1]. Permafrost accounts for 24% of the land area at northern latitudes [2]. The global reserve of permanently frozen SOC is estimated at 1700 Pg C, twice that of the atmosphere [3]. A number of climate models predict that northern regions (above 60°N) will experience climatic changes, in particular warmer temperatures and higher precipitation [4, 5]. These changes have environmental effects such as permafrost thaw [6], altered hydrology [7], erosion of palsa mires [8], vegetation change such as tree line and shrub expansion [9], and increasing organic matter loading, especially as dissolved organic carbon (DOC) in rivers [10].

An implication of permafrost thaw is that the active layer in soil deepens, such that previously frozen SOC becomes susceptible to microbial degradation, which releases CO_2_ and CH_4_ [11, 12]. Moreover, thawing permafrost promotes the release of old carbon into rivers as DOC [13], where it can be further degraded into CO_2_. This lateral transport of carbon via DOC could be expected to further increase in future climate change scenarios of increasing precipitation and increased hydrological flows [14, 15].

Climate warming causes phenological changes such as a shift in the growing season start and end (extended span), an increase in net primary productivity (NPP), and increased forest cover [16, 17]. This increase in biomass allows terrestrially derived organic matter to become an increasingly important lateral source of carbon at the catchment scale. Therefore, understanding the release of DOC from northern catchments, which have a store of permafrost, together with increased terrestrial productivity is a major concern in biogeochemical research.

Strong evidence for altered land-water coupling in the carbon cycle at northern latitudes e.g. Scandinavia, Canada, and Germany is the phenomenon of brownification of inland waters [18–20]. The change in water colour to brown is mainly due to an increase in terrestrially-derived dissolved humic substances [21], typically being reflected in increased DOC concentrations [22–24]. Other causes of brownification that have been suggested in the literature include changes in vegetation, declining sulphate deposition, an increase in precipitation, and land use change [25–28]. Browner waters limit light penetration in water bodies, thus decreasing the primary production and affecting secondary production of aquatic fauna [29, 30]. Brownification also leads to increased remineralization of DOC in inland waters, and thus increased fluxes of CO_2_ to the atmosphere [31, 32].

Although the contributions from inland waters to gaseous carbon in the atmosphere are increasingly acknowledged in global carbon cycle studies, the lateral export of dissolved organic carbon (DOC) at high latitude regions remains an emerging area of research [33]. Lateral interactions, e.g. permafrost thaw, have the potential to increase the carbon release to the atmosphere from high latitude regions, possibly turning landscapes from carbon sinks into sources [34, 35]. Further research is thus needed to reach a conceptual understanding of the controls on DOC release from catchments, particularly in subarctic regions which are strongly affected by climate change. One way to do this is to investigate the coupling of landscape components in biogeochemical cycles such as carbon at varying spatio-temporal scales [36, 37]. Another step is to refine regional estimates using direct measurements and topographical data. Research in northern Sweden has begun to do this by incorporating lateral carbon fluxes in models through the use of distributed hydrological modeling, and mixing-models using geological landscape elements [38, 39].

Multiple studies in Fennoscandia have used the Normalized Difference Vegetation Index (NDVI) to quantify changes in vegetation productivity and linked them to DOC concentration in recipient waters [27, 39, 40]. Larsen *et al*. [41] used the NDVI as a proxy for vegetation density in their study of lake organic carbon concentrations in Norway. They found that vegetation density was a strong positive predictor of lake organic carbon concentrations. Moreover, in a continental (Europe) scale study of soil pore waters, a positive correlation between the NDVI and DOC concentration in broadleaf forests was found [42]. However, there is need for further studies to understand how NDVI and other catchment properties regulate DOC in the subarctic, and whether or not *a priori* knowledge about land cover type (mire, forest etc.) needs to be included in order to build a predictive model for sub-catchment DOC variations.

In this study we aim to determine (a) the relationship between the NDVI and DOC at subcatchment level, (b) the degree to which other generic catchment properties (elevation, slope) explain DOC, and; (c) whether or not additional land cover variables representing the local vegetation (mire, forest, lake, tundra) need to be included in predictive models for DOC delivered into rivers. We hypothesize that subcatchments with higher terrestrial productivity generate higher DOC concentrations in the subarctic catchment Stordalen, Sweden. Temporally, we anticipate a time lag in the DOC response to changing productivity. This arises as we assume that the effect of productivity during a given year will be captured by DOC measurements performed throughout the growing season and subsequent period, ending at early snowmelt of the following year.

## 2. Materials and methods

### 2.1. Ethics statement

Data used in this study was collected with permission from the Abisko Scientific Research Station (ANS) by Olefeldt et al. [43] and Lundin et al. [44] who supplied us with the data for Stordalen. Endangered or protected species were not used in this research.

### 2.2. Site description and experimental design

**Fig 1:**
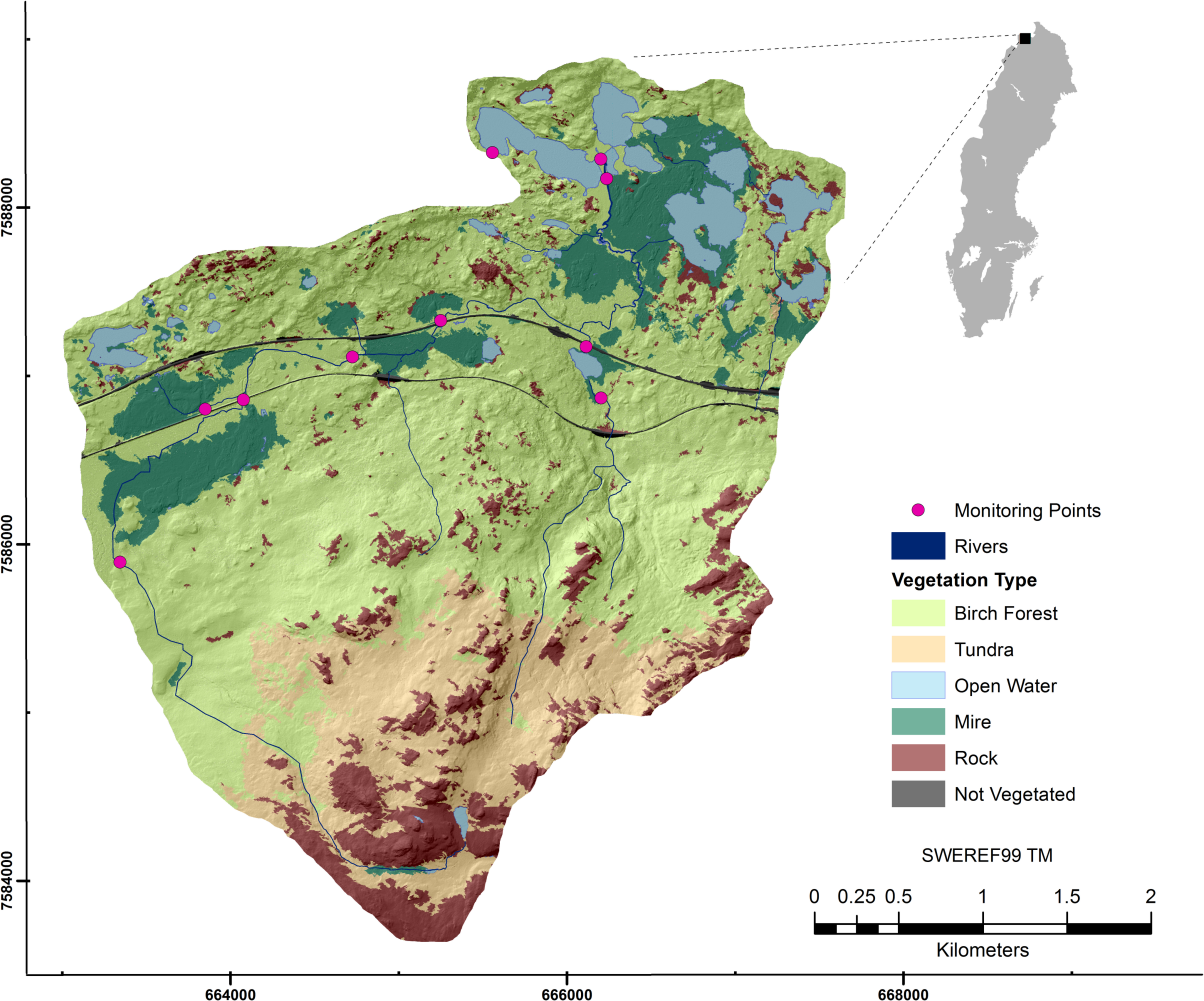
Vegetation map of Stordalen catchment, Sweden. Map data sources [38, 49].

Stordalen catchment (15 km^2^; 68°21’N, 19°03’E) is located in northern Sweden, 10 km east of the village of Abisko (see Fig 1). It lies in the subarctic climatic zone with the coldest temperatures in February and the warmest in July [45]. Mean annual temperature (MAT) for the period 2000 to 2009 was between 0.6° and 0.7°C [46, 44]. This is higher than at the start of the 20^th^ to early 21^st^ century, where MAT temperatures were between -0.7°C and -0.6°C [47, 48]. Mean annual precipitation lies between 304 mm (in 2003) to 308 mm (in 2006) [47, 34].

The topography in Stordalen is characterized by a gradient from high elevation (≈800 m.a.s.l.) in the southern part to low lying topography (≈360 m.a.s.l.) in the north. The upper (southern) catchment has steep slopes consisting of igneous and metamorphic rock outcrops and tundra, with patchy lakes and mires. The streams in this part of the catchment are single channels which are fed by precipitation and snowmelt. Lower catchment streams are dispersed and drain into larger lower lying lakes. Abundance of lakes and peatland mires in the lower part of the catchment is facilitated by gentle slopes, moraine and peat soils. The inflection point of the catchment is marked by birch forest (*Betula pubescens spp*) and further towards the north, by the emergence of mires and lakes [38].

Stordalen mire is characterized by three land cover types: palsa, sphagnum, and eriophorum, respectively [50]. These land cover types are found where permafrost degradation has taken place in the catchment.

We selected ten nested subcatchments, defined by 10 monitoring points in Stordalen catchment, to achieve the aims of this study. Each subcatchment has a mixture of land cover types i.e. mire, birch forest, lakes etc. shown in Fig 1. First, single relationships between monthly averaged DOC concentration and variables that reflect catchment productivity and main geographical features (slope, elevation) were tested. We then built multiple linear mixed effects regression (LMER) models to explore how DOC predictability might be improved when land cover variables are included. Finally, results from the LMER models were extrapolated and applied to the whole Stordalen catchment, in order to visualize the contribution to surface water DOC from each pixel in the catchment.

To quantify the vegetation productivity we used the NDVI as a proxy for net primary production (NPP) i.e. vegetation productivity. The NDVI has been used to represent NPP in the field [51–54], particularly in Light/Rain Use Efficiency (L/RUE) models based on the Eq 1 below from Yengoh et al. [55]:

NPP=f(NDVI,PAR,fPAR,aPAR,LAI) (1)

We used the NDVI from the easily accessible Moderate Resolution Imaging Spectroradiometer (MODIS) NDVI data as an indicator of NPP in Stordalen.The NDVI is a widely used and relevant indicator used in a plethora of land use change studies and finds use in a number of northern latitude studies e.g. [41]. It was selected as a quick broad scale assessment of terrestrial productivity.

### 2.3. Field sampling and laboratory analysis

**Fig 2:**
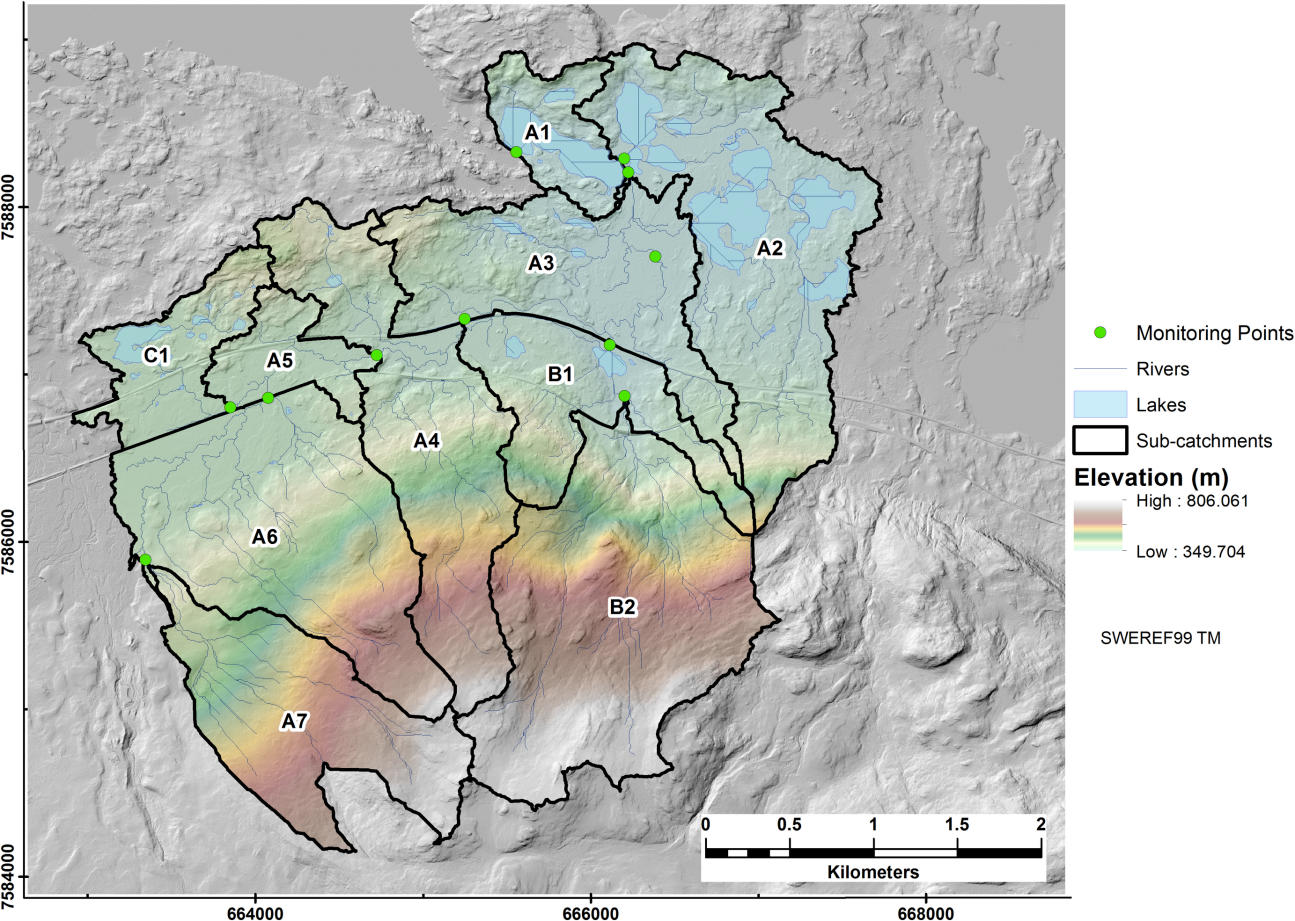
Nested subcatchments in Stordalen (borders in black) delineated using the D8 algorithm.

Water monitoring data was obtained for 2007 and 2008 from Olefeldt et al. [43,56]. For the period 2009 to 2011 data was provided by Lundin et al. [44]. Stream grab samples together with samples from in situ autosamplers were taken and analyzed for DOC (in mg L^-1^), water temperature (°C), pH, total nitrogen, and conductivity. Water samples were taken at a monthly interval using an acid washed polythene bottle which was refrigerated prior to analysis [44]. The autosamplers (Hach Sigma 900) collected water on a 3 to 6 days cycle [43]. The samples underwent filtration using a 0.45 μm filter followed by acidification with HCl. The samples were then inserted in the Shimadzu TOC-V CPH analyser to determine the DOC content [44]. We utilized the 10 monitoring points by Olefeldt et al. [56] as a basis for sub-catchment delineation, as shown in Fig 2.

Stream stage was measured 2007–2008 at hourly intervals using Odyssey capacitance water level probes [55]. Hourly direct discharge (m^3^/s) measurements were approximated using the velocity-area method, using an Aqua Data Sensa Electromagnetic Current Meter to measure velocity [56]. Stream stage and discharge data were used to generate rating curves which were then used to derive hourly discharge for the snow free period [56]. Since discharge data was available for two years only (2007–2008) and since the number of DOC measurements varied between sites and years, complete DOC exports were not possible to calculate in this study. Therefore, the DOC concentration was used as the main carbon release indicator in the catchment as there was a longer record of concentration data than of discharge data.

To analyze relationships between DOC and single catchment property variables (aims [a] and [b] in Section 1), the discharge weighted DOC (DOC_qwc_) was used as a response variable [57] providing an integrated representation of the overall DOC delivery to recipient downstream waterbodies. The DOC_qwc_ was calculated for each subcatchment based on DOC concentration (C) and discharge (Q) (Eq 2) for the period 2007–2008 when flow data was available.

$$DOC_{qwc}=\frac{\sum_{i=1}^{n}C_{i}Q_{i}}{\sum_{i=1}^{n}Q_{i}}$$ (2)

The DOC_qwc_ provides a representation of the mean DOC concentration in all runoff that is received by downstream water bodies such as lakes [58]. Assuming that areal discharge is similar for the different sub-catchments, DOC_qwc_ also serves as a proxy for spatial differences in total DOC export.

### 2.4. Data preparation

Elevation data were obtained from the Swedish National Land Survey (Lantmäteriet). The 2 m GSD-Höjddata, grid 2+ is a publicly available, licensed, ASCII format, DEM downloaded in the projection SWEREF99 [49]. Using the ArcMap® Hydrology Toolbox, rivers and subcatchments were assigned using the single flow direction (SFD) D8 algorithm. Artefacts in the landscape such as sinks and anthropogenic structures, e.g. roads and railways, were breached to replicate the natural flow of water within the catchment.

The moderate-resolution imaging spectroradiometer (MODIS) was used as it provided NDVI (MOD13Q1) 16 day composites at 250 m resolution. MODIS data was downloaded for the years 2006 to 2012 from the Oakridge National Laboratory Distributed Archive Center [59]. The data was downloaded in the geographic coordinate system WGS84 and projected to SWEREF99. The NDVI is a unit less vegetation index values ranging between -1.0 and 1.0 (maximum greenness) using the general equation (Eq 3) below.

$$NDVI=\frac{NIR-red}{NIR+red}$$ (3)

The annual seasonal mean NDVI of each subcatchment was extracted using Zonal Statistics as Table in Spatial Analyst Tools in ArcMap 10.2.2. This process was repeated for each NDVI TIFF file for the period 2007–2011, for the months of May to September, to yield the mean of the two 16 day composite NDVI per subcatchment and month. The Slope tool (ArcGIS) was used to extract the degree of slope for the catchments from the DEM. Mean slope for each subcatchment was again extracted. Mean elevation (m.a.s.l) and land cover classes (mire, forest, tundra, slope, elevation) [38] per subcatchment were extracted using the same zonal statistics method.

### 2.5. Statistical analysis

The correlation between the NDVI, DOC (arithmetic mean per year), and DOC_qwc_ (flow-weighted mean per year) was tested using simple linear regression [60]. Relationships between DOC and additional catchment variables (NDVI, mire, forest, lake, tundra, slope, and elevation) were tested in the same way using SPSS. To eliminate redundancy in input variables, the tundra class was removed since tundra cover is equal to the area not covered by mire, forest or lake.

A weakness that arises with calculating individual annual mean DOC values for the 10 respective subcatchments is that the number of data points becomes low, leading to low statistical power. Hence, in the final analysis part (aim [c]), more robust predictive models for DOC (LMER) was applied by using DOC data for multiple time points per year (monthly averages). LMER models for DOC were built using the statistical package “*lm4”* in R® with NDVI, slope, elevation and land cover (forest, mire, lake) as predictor variables, and time (months since start of measurements) as random effect variables. The annual mean NDVI was extracted per subcatchment for the period May to September. The percentage of land cover per subcatchment was calculated and used to determine the cumulative contribution into or from a subcatchment, e.g. subcatchment A1 receives the sum of all subcatchments as it is the outlet for Stordalen. This approach ensured that the land cover in the input tables for the LMER was representative of the class for the catchment and not just the subcatchment level.

A LMER model was first established by entering all explanatory variables. Then backwards elimination was carried out by manually removing one explanatory variable at a time (in all possible combinations) based on the p-value, to determine whether or not it could be removed without significantly changing the model and losing predictability. An ANOVA test was performed at each exclusion step for cross model comparison. To obtain conditional (complete model) and marginal R^2^ estimates (R^2^c and R^2^m, respectively), the package *MuMIn* was used, while significance of coefficients (fixed effects) were tested with the package *lmerTest*. The results of the LMER models were classified into six classes using the geometrical interval in ArcGIS.

## 3. Results

Ten subcatchments were delineated. The subcatchments had good representation of the main flow paths and associated lakes in Stordalen (Fig 2). A distinct pattern of lakes at lower elevations, i.e. below the catchment inflection point, was observed with rivers at higher elevations typified by straight and narrow channels.

### 3.1. NDVI-DOC relationship

**Fig 3:**
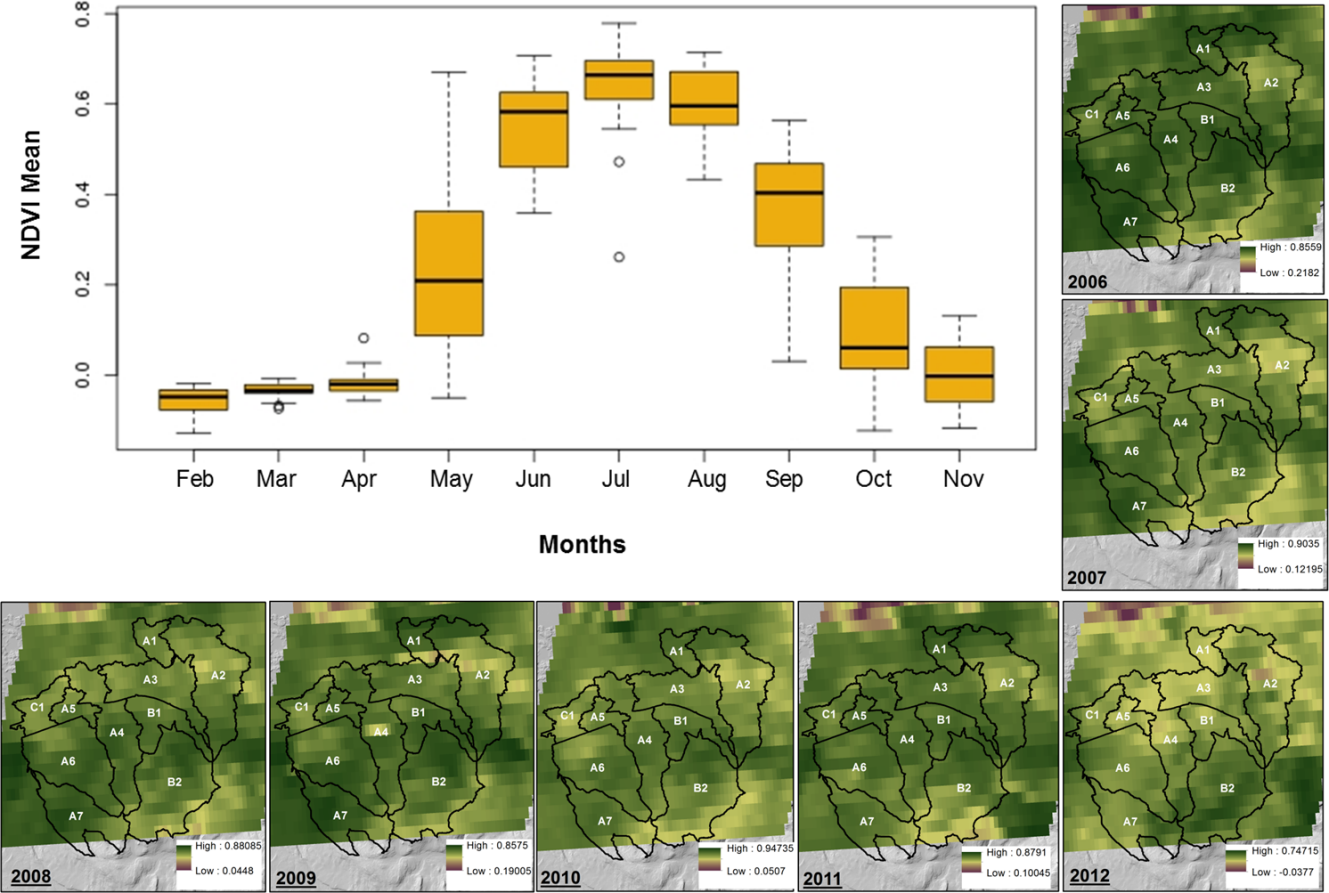
Boxplot of mean monthly NDVI values for the 16-day composites at 250 m resolution for the years 2006–2012 (adapted from ORNL [59]). Annual mean NDVI for the catchment is shown with subcatchment boundaries in black overlain to annual variation across subcatchments.

Peak NDVI values were consistently observed in July as shown in Fig 3. NDVI changes from 2006 to 2012 showed that the eastern periphery and southern subcatchments e.g. A2, B2, A7, and C1 experienced the most change in NDVI. Interior subcatchments showed little change in annual comparisons.

To determine whether the NDVI had an effect on the mean annual DOC concentration (n = 30) and mean annual DOC_qwc_ (n = 20) in the catchment, a simple linear regression for the years 2007–2009 was performed with the significance threshold set at 0.05. The DOC yielded an r-squared of 0.137 (p = .044) for DOC and the DOC_qwc_ an r-squared value of 0.114 (p = .146).

**Fig 4:**
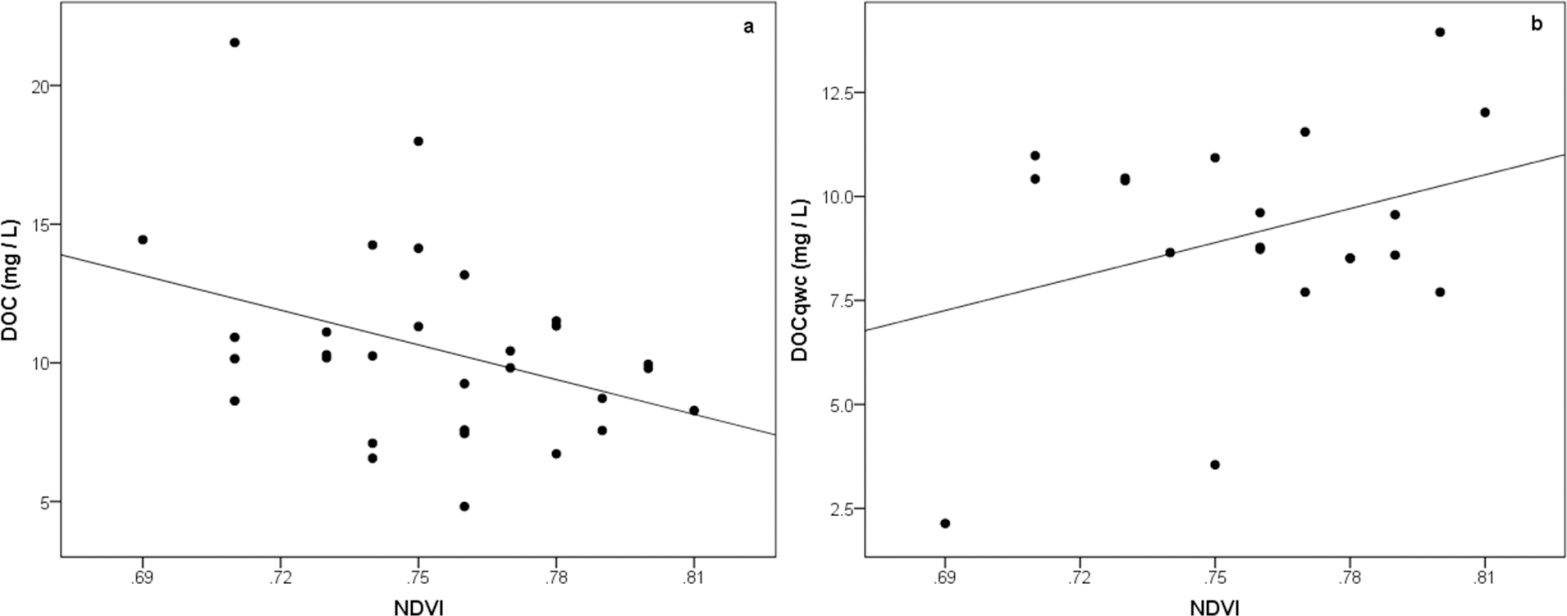
**Scatterplot of (a) mean annual dissolved organic carbon (DOC) concentrations and (b) flow-weighted mean annual DOC against NDVI per subcatchment.** The DOC regression line shows Y = -41.765x + 41.971, R^2^ = 0.137 and for DOC_qwc_ Y = 0.0042x + 0.7208, R^2^ = 0.114.

The DOC vs NDVI (Fig 4A) had a statistically significant relationship, whilst the DOC_qwc_ vs NDVI (Fig 4B) did not. However, the trendline for DOC_qwc_ was positive which could have supported our hypothesis had the p-value been lower. Subcatchments with high NDVI yielded low DOC concentration values.

**Fig 5:**
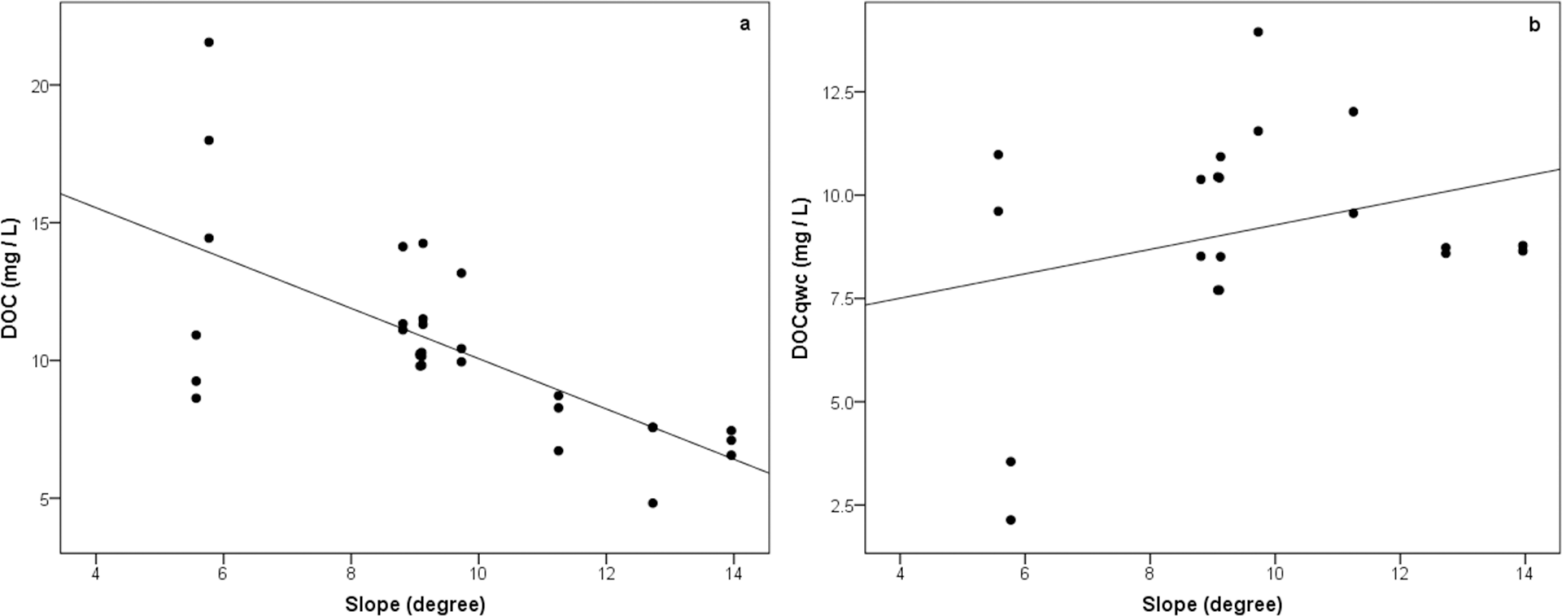
**Scatterplot of (a) mean annual dissolved organic carbon (DOC) concentrations and (b) flow-weighted mean annual DOC against Slope.** The DOC regression line shows Y = -0.5047x + 14.816, R^2^ = 0.461 and for DOC_qwc_ Y = 0.2789x + 6.9655, R^2^ = 0.082.

Simple linear regression was again performed to assess the extent to which slope can explain the observed DOC fluctuations. Results (Fig 5A) yielded a negative significant relationship with an r-squared value of 0.461 (p < .001) for DOC.

The DOC_qwc_ had a non-significant relationship with slope (Fig 5B), producing an r-squared of 0.082 (p = .220).

**Fig 6:**
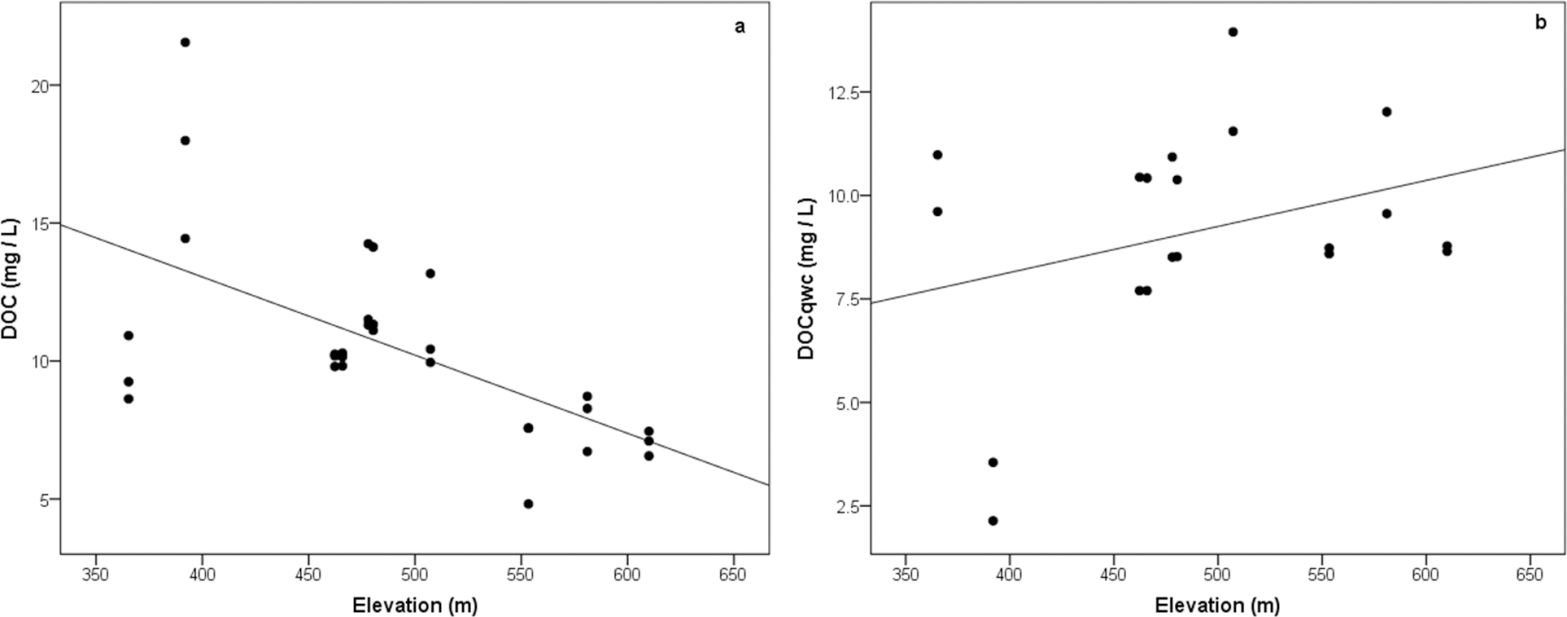
**Scatterplot of (a) mean annual dissolved organic carbon (DOC) concentrations and (b) flow-weighted mean annual DOC against Elevation per subcatchment.** The DOC regression line shows Y = -13.209x + 628.36, R^2^ = 0.374 and for DOC_qwc_ Y = 8.853x + 408.67, R^2^ = 0.099.

The process was repeated to assess influence of elevation on the DOC. The linear regression results for DOC (Fig 6A) yielded a significant negative relationship with an r-squared value of 0.374 (p < .001) for DOC concentration.

The DOC_qwc_ had a non-significant relationship with the NDVI (Fig 6B), producing an r-squared of 0.099 (p = .178).

In our second objective we sought to determine the extent to which catchment properties (i.e. land cover) influences DOC in Stordalen. Earlier in Section 3.1 the NDVI, slope, and elevation were compared against DOC and DOC_qwc_. However, the NDVI, slope and elevation are not representative of all generic (sub) catchment variables e.g. vegetation type. A Pearson’s Correlation was thus performed, testing for significance at the 0.05 and 0.01 levels between land cover classes (mire, forest, lake, tundra, slope and elevation) and DOC for all available years. The results of the correlation (Table 1) show that DOC has positive significant correlations with the mire, NDVI, and forest. The DOC had significant negative correlations with slope and tundra.

**Table 1:** Correlation matrix of average monthly DOC concentration with fixed land cover proportion per subcatchment per year together with the seasonal maximum NDVI. Slope (n = 50), Mire (n = 50), Forest (n = 50), Lake (n = 9), NDVI (n = 50), Elevation (n = 50), and DOC (n = 218).

	DOC	NDVI	Slope	Mire	Forest	Lake	Tundra	Elevation
DOC								
NDVI	0.354**							
Slope	-0.483**	-0.346**						
Mire	0.586**	0.316**	-0.923**					
Forest	0.282**	0.408**	-.169*	.144*				
Lake	0.098**	-0.031	-0.683**	0.594**	-0.575**			
Tundra	-0.419**	-0.227**	0.907**	-0.934**	-0.002	-0.748**		
Elevation	-0.051	-0.366**	-0.189**	0.203**	-0.082	0.216**	-0.239**	

*. Correlation is significant at the 0.05 level (2-tailed).

**. Correlation is significant at the 0.01 level (2-tailed).

The NDVI had a positive and significant correlation with forest, DOC and mire. Conversely, the NDVI has a negative and significant correlation with slope, elevation and tundra. NDVI values for the years 2007 to 2011 were consistently low for subcatchment B2 which has steep slopes, tundra, rock outcrops, and high elevation values. Subcatchment A2 also had low NDVI values, second to B2, during this period. High NDVI values were found in subcatchments A1 and A5 in the five year period. Subcatchments A6 (2008–2009), A7 (2010) B1 (2007), and C1 (2011) had the third highest NDVI values in their respective years.

Slope had a positive significant correlation with tundra (.907) only. In contrast, tundra showed significant negative correlation with mire, lakes and DOC. Notably; slope had negative and significant relationship with all land cover classes, with the exception of tundra.

Mires had the strongest, positive and significant correlation with lakes, DOC and NDVI. However, they (mires) showed a strong negative correlation with tundra (-.934) and slope (-.923). Lakes had a positive and significant relationship with mires.

### 3.2. Linear mixed effects models

The backwards elimination procedure resulted in three explanatory models for Stordalen catchment (summarized in Table 2). In all three cases, all of the coefficients were significant (p < 0.05). In the exclusion process, removal of specific land cover variables (i.e. mire, forest, lake), yielded a poorer fit. Hence, these variables were kept in all models. The tundra class was not included in this step as it constitutes what is not covered by mires, forest and lake land cover classes.

**Table 2:** LMER model equations for DOC concentration Stordalen catchment. Models are ranked by marginal and conditional R squared values. Slope (n = 50), Mire (n = 50), Forest (n = 50), Lake (n = 9), NDVI (n = 50), Elevation (n = 50), and DOC (n = 218).

Model	Equation	AIC	BIC	R^2^m	R^2^c
**1**	*DOC*_*M*1_ = *Slope*(−1.5) + *Mire*(32.5) + *Forest*(−46.5) + *Lake*(−95.3) + 52.9	984.6	1008.2	0.46	0.75
**2**	*DOC*_*M*2_ = *NDVI*(31.1) + *Mire*(50.7) + *Forest*(−21.3) + *Lake*(−44.2) + 1.4	993.3	1017.0	0.43	0.77
**3**	*DOC*_*M*3_ = *Elevation*(−0.0059) + *Mire*(55.4) + *Forest*(−13.0) + *Lake*(−40.9) + 17.3	997.2	1020.9	0.42	0.73
**Full**	*DOC_Full_* = *Slope*(−1.5) + *NDVI*(5.8) + *Elevation*(−0.004) + *Mire*(35.1) + *Lake*(−95.5) + *Forest*(−48.1) + 51.9	1044.0	1074.4	0.46	0.61

The Akaike Information Criterion (AIC) and Bayesian Information Criterion (BIC) are ways to measure model fit (the lower the AIC and BIC the better the fit). The AIC and BIC (n = 218) are presented together with the marginal (R^2^m) and conditional (R^2^c) r-squared values of the models.

In all models, the mire class stands out as a positive DOC explanatory variable in Stordalen. In Model 2, the NDVI serves as a secondary significant positive predictor of catchment DOC. With the exception of the full model, Model 2 is the only model that includes the NDVI. Similarly, elevation and slope appear only in Model 3 and Model 1, respectively. The full model, which provides a comparison with the three models, had the lowest marginal and conditional r-squared values and the highest AIC and BIC values.

The significance and correlation tables of the full model were exported to provide the overall understanding of the LMER results (Table 3 and Table 4). Results of the fixed effects (Table 3) showed that significant predictors were slope, mire, lake and forest classes.

**Table 3:** Significance of fixed effects terms of the full model. Slope (n = 50), Mire (n = 50), Forest (n = 50), Lake (n = 9), NDVI (n = 50), and DOC (n = 218).

	Estimate	Std. Error	df	t value	Pr(>|t|)	Signif.
Intercept	51.89	12.90	208.69	4.02	0.00	***
Slope	-1.46	0.46	206.59	-3.20	0.00	**
NDVI	5.83	5.66	207.39	1.03	0.30	
Elev	0.00	0.00	209.67	-1.51	0.13	
Mire	35.10	8.01	205.93	4.38	0.00	***
Lake	-95.50	17.83	205.81	-5.36	0.00	***
Forest	-48.05	11.43	205.80	-4.20	0.00	***

Signif. level: 0.001 ‘***’ 0.01 ‘**’

**Table 4:** Correlation of fixed effects for the full model. Slope (n = 50), Mire (n = 50), Forest (n = 50), Lake (n = 9), NDVI (n = 50), Elevation (n = 50), and DOC (n = 218).

	(Intr)	Slope	NDVI	Elev	Mire	Lake	Forest
Slope	-0.945						
NDVI	-0.361	0.145					
Elev	-0.196	0.010	0.464				
Mire	-0.646	0.805	0.021	-0.082			
Lake	-0.930	0.923	0.095	-0.026	0.561		
Forest	-0.907	0.895	-0.003	-0.046	0.550	0.966	

The NDVI and elevation were not significant predictors of DOC in the full model as shown in Table 3. They do emerge in Models 2 and 3 which have higher R^2^m values when compared to the full model. Lakes had the highest negative trend whilst mires showed the opposite. In the full model, forest and slope terms were negative, whilst the NDVI term was positive. The negative pattern by forest and slope was mantained in Models 1 to 3 with elevation being negative where it appeared as a fixed effect in models.

### 3.3. Spatial representation

We extrapolated the equatons in Table 2 to each pixel of the study area to visualise what the models suggest about how each part of the Stordalen catchment contributes to surface water DOC.

**Fig 7:**
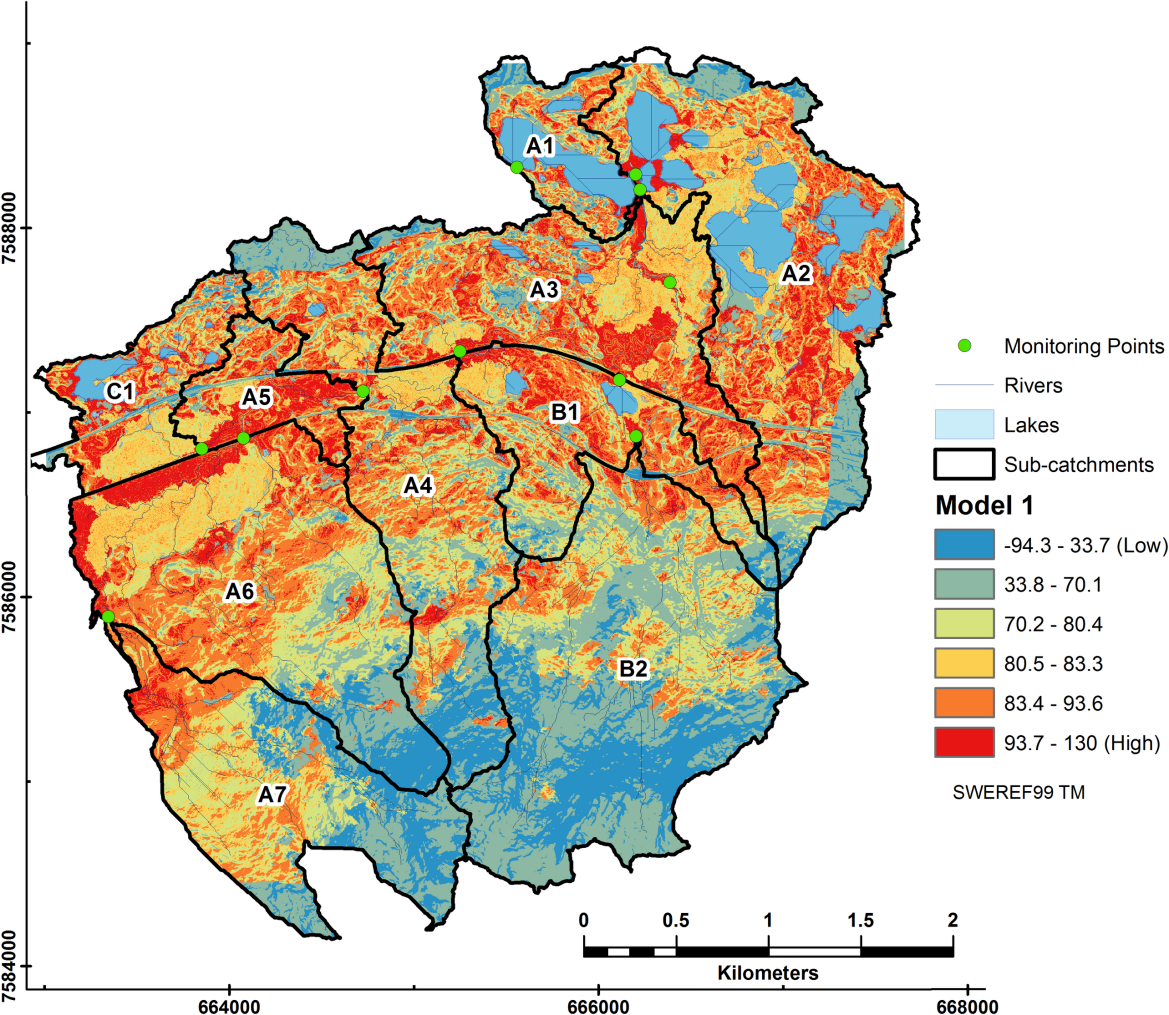
Relative DOC contributions from subcatchments to riverine DOC in Stordalen from Model 1. Coefficients and the intercept (52.9) were extrapolated from LMER Model 1 to each pixel using the fixed terms of the model equation. DOC loss (denoted in blue) occurs in lakes and where steep slopes occur with forest land cover.

Model 1 (Fig 7) shows that lakes, found largely in low lying subcatchments A3 to A1, contribute the least to DOC export in Stordalen. Another region were DOC contribution was minimal was where there were steep slopes and a mixture of omitted classes such as tundra and outcrop (e.g. upper B2, A7, A4 and A6).

Interestingly; locations surrounding mires also had high contributions towards DOC. These areas were characterized by low slope angle and forest cover (e.g. A6 and A5).

**Fig 8:**
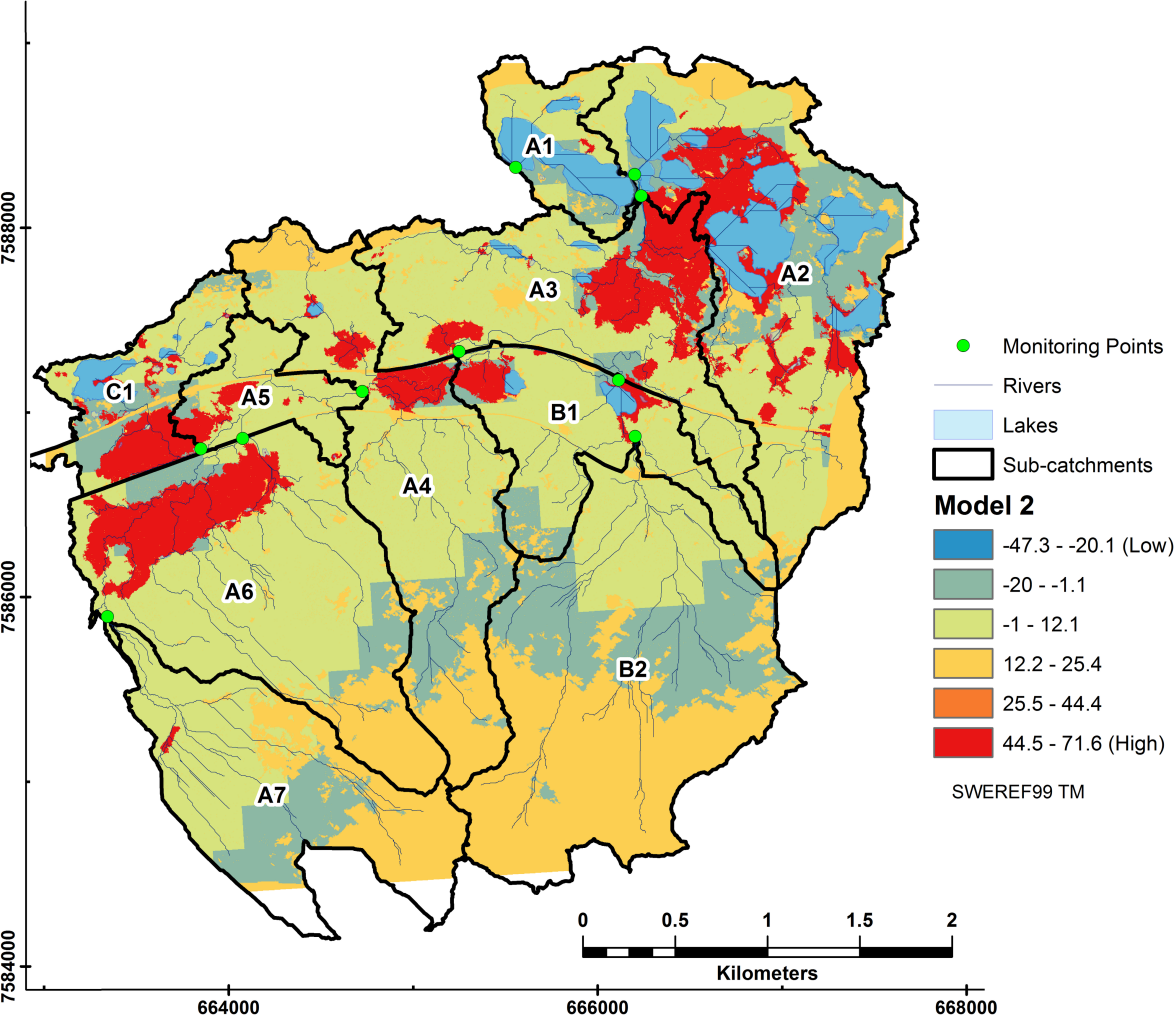
Relative DOC contributions from subcatchments to riverine DOC in Stordalen from Model 2. Coefficients and the intercept (1.4) were extrapolated from LMER Model 2 to each pixel using the fixed terms of the model equation. DOC loss (denoted in blue) occurs where lakes and forest land cover dominate.

In Model 2 (Fig 8) we found that forest cover lowered the effect of high NDVI values with its negative sign in the LMER equation, thus accounting for the large spread of the green (-1 to 12.1) grouping in Fig 8.

The tundra class (upper regions of subcatchments A6, A7, and B2) was included by omission, i.e. the area not covered by any other land cover classes. It shows up as moderate to low values.

**Fig 9:**
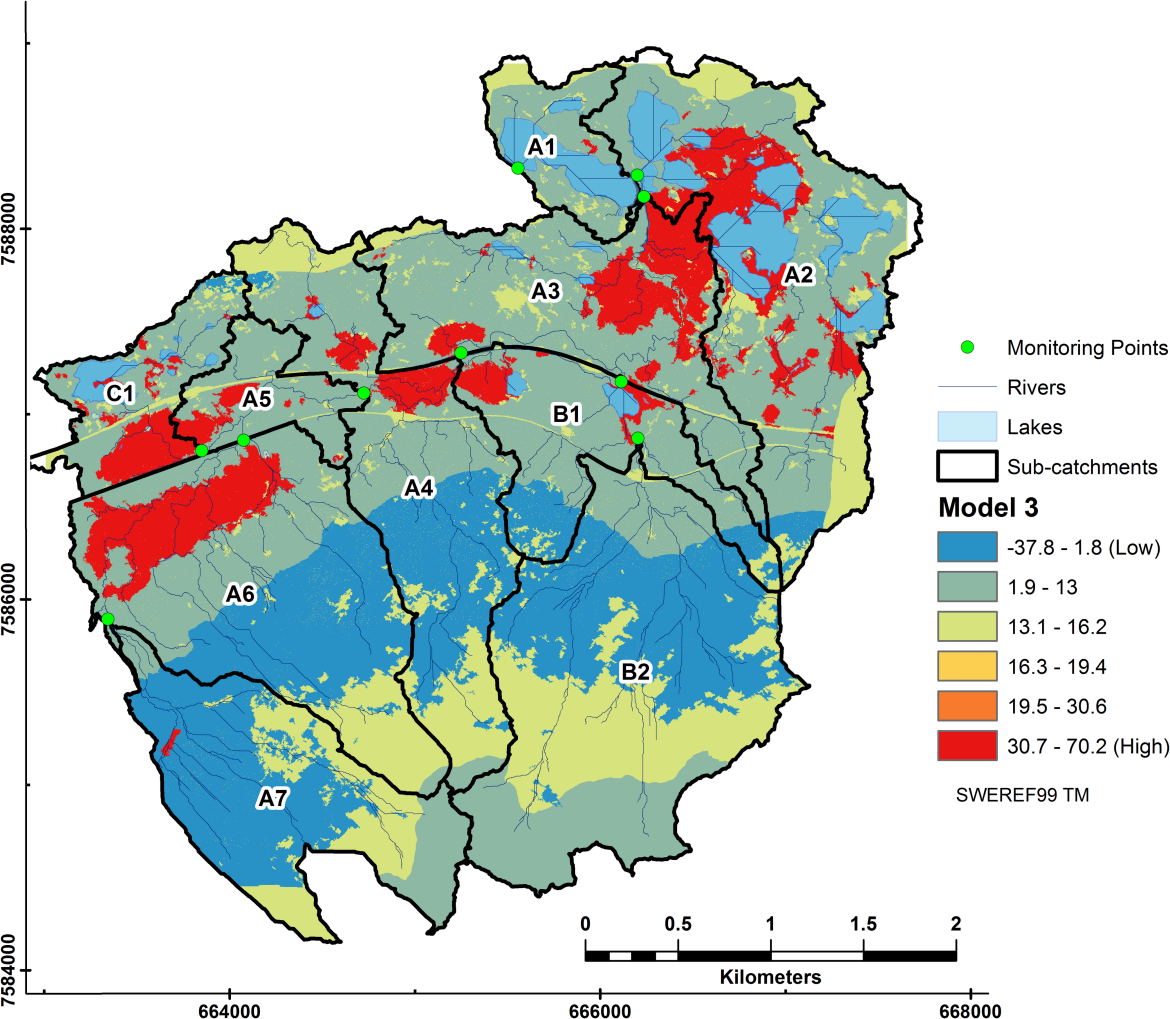
Relative DOC contributions from subcatchments to riverine DOC in Stordalen from Model 3. Coefficients and the intercept (17.3) were extrapolated from LMER Model 3 to each pixel using the fixed terms of the model equation. DOC loss (denoted in blue) occurs where lakes and high elevation combine with forest land cover.

Mire is the only class in Model 3 that does not have a negative coefficient and shows up as the strongest contributor of DOC in the catchment (Fig 9). Model 3 lacks intermediate classes due to the fixed effects having negative values associated with them (mires excluded). This is already evident in Table 2 where the mire class is the only variable with a positive coefficient.

Steep to moderately steep areas that have forest cover, shown as the dark blue band in subcatchments A7, A6, A4 and B2, stand out in this model as they together with lakes, have the lowest contribution to DOC.

## 4. Discussion

Our hypothesis was to show that increased terrestrial productivity, represented as the NDVI, generates higher DOC concentrations in the Stordalen catchment. Using simple linear regression in our first objective, the NDVI showed a significant negative relationship with DOC concentration. DOC_qwc_ was marginally significant with the NDVI. This was interpreted as a difference in scale that the two DOC indicators capture. More specifically, the flow-weighted mean DOC better represents DOC concentration in the entire export. Thus, the NDVI did not appear to show total export. At the local level, high DOC values can be found where NDVI values are low. Low NDVI values coincided with land cover classes such as mires. The NDVI had its highest significant positive correlations with mire and forest land cover classes. Birch forest, having the largest land cover type in Stordalen, and being detected by the NDVI, contributes organic matter litter in the subcatchments thereby enriching the DOC pool.

LMER tests were further carried out to tease out the NDVI-DOC relationship in Stordalen adding land cover variables into the analysis. The NDVI had a positive but secondary regulatory effect on DOC as it was a predictor variable in Model 2. Furthermore, the NDVI together with elevation were non-significant in the full model when compared with other fixed effect terms. Mires, in all models, were the strongest positive determinant of DOC. These findings of the NDVI-DOC relationship show that the NDVI on its own is not a good indicator of the impact that terrestrial productivity has on DOC in recipient waters, probably because land cover variables (especially mires) exert the primary control on DOC and our window of the NDVI analysis in terms of years was brief.

Our result that mires are key predictors of DOC rich subcatchments in Stordalen supports the findings of other studies that reported them to have an additive effect and constitute one of the key controls of DOC export and concentration in catchments [61–63]. In the subarctic, it has been found that forests contribute to DOC export but spatially their contribution to DOC export is exceeded by mires [64]. Thus, the presence of mires reduces the NDVI-DOC relationship at the landscape level.

Besides the mire class, other catchment properties such as slope, elevation, tundra, and forest (birch) were compared against DOC. A significant negative relationship between DOC concentration and slope was found. This adheres to catchment land cover distribution in Stordalen as localities where steep slopes dominate, are largely covered by tundra and rock outcrops, while gentle near flat topography is host to mires. This assertion is supported by the significant positive slope and tundra correlation value of 0.907. Slope also had a strong negative, near linear, and significant correlation with the mire class. This reinforced flat areas, which are host to mires, as DOC hotspots in Stordalen. In the LMER models slope was a significant negative and primary predictor of DOC. Low DOC concentration at steeper slopes in the catchment is congruent with hydrological solute transfer by gravity i.e. quick downstream transfer. The positive non-significant relationship DOC_qwc_ had with slope indicates that at the broader scale, catchments that have steep slopes will efficiently transport DOC to a recipient water body at the end of the system. Thus, increasing DOC values with slope can be related to flushing. Slope as a key predictor of DOC is not limited to Stordalen as slope has been observed as a strong predictor at continental level [65].

Elevation was a tertiary predictor of DOC in the study where it paired best with mire, forest, and lake classes in Model 3. However, as a fixed effect term it was non-significant. In the simple linear regression, elevation had a negative relationship with DOC concentration. This is a function of catchment land cover type, where high elevation zones are characterized by rock outcrops and tundra. Additionally, in the discontinuous permafrost zone, areas underlain by permafrost zones (i.e. SOC enriched zones) can be limited by topography in the subarctic [66]. In Stordalen, this manifests as the lowest elevation areas being host to the peatland mire.

Of all the variables used in the correlation matrix, the strongest significant positive relationship with DOC was the mire class. Additionally, the mire class was consistently positive in all our LMER models. However, mires also had significant positive correlations with lakes and the NDVI. The influence of mires begins to explain the modest correlation value between the NDVI and DOC. Even though mires have low NDVI values, their role in subsurface contributions of DOC cannot be ignored.

In contrast to mires, lakes in all LMER models were found to have a strong negative effect on DOC. This observation suggests that likely no major autochthonous production of DOC occurs in these lakes, but rather lakes serve as transformation zones where DOC is lost. In Stordalen, lakes had a significant positive correlation with mires. This arises due to spatial arrangement in the catchment i.e. lakes and mires are often adjacent to each other or within the same elevation zone. Conversely, tundra and slope had significantly negative relationships with lakes.

As expected, birch forest had its highest positive and significant correlation with the NDVI followed by DOC. In the LMER models, the birch forest had an antagonistic effect on DOC. Generally, the proportions of mire and forest classes are used to predict DOC export in the boreal region [39]. However, the forest class in this case was negative. Although a primary predictor of DOC in Stordalen, the negative sign of forest in all the LMER models suggests that we assess the robustness of the NDVI as a proxy, particularly weaknesses in the manner in which it deals with saturation [67]. The NDVI has been shown to perform weakly in dense conifer forests in the boreal region [68]. These factors compounded by our assessment period (May–Sept), begin to explain the negative sign of the forest land cover class, even though it is the largest class in the catchment.

Significant negative relationships with DOC were found with topographic indicators slope and tundra. Accordingly, in Stordalen higher DOC can be expected where there are mire and forest land cover classes. Periphery subcatchments associated with tundra and rock outcrop classes (e.g. A7, B2) had low NDVI values in Fig 3. Similarly, maps of DOC distribution show that the upper zones of periphery subcatchments contributed least to DOC.

The potential role of groundwater cannot be overlooked in influencing DOC concentrations in Stordalen and our findings. Subcatchments that receive regional groundwater inputs (e.g. B1, B2) had lower DOC concentrations as a result of higher runoff and electrical conductivity [69]. Groundwater, a variable that causes variability in DOC concentration, is not accounted for in our models. Hence, our DOC predictions are limited in this regard.

The DOC_qwc_ was incorporated to give a broad picture of DOC dynamics from the catchment. In some cases such as slope and NDVI, the DOC_qwc_ provided estimates of long term DOC interactions. For elevation DOC_qwc_ did not provide meaningful results in our analysis. However, what became evident with DOC_qwc_ results was their dependency on dilution. In the future, a longer flow record and scaling may show better spatio-temporal differences, which could benefit our DOC_qwc_ analysis.

## 5. Summary and conclusion

As inland waters are becoming more important in global carbon studies, it is important to understand the role that the surrounding landscape contributes to the measured DOC. Rivers are dynamic systems that, in contrast to lakes, flow through different land cover classes and media. They receive inputs from subsurface and overland (infiltration and saturation excess) flow, as well as precipitation, which change sometimes at fast rates. Climate change predictions show increases in vegetation. High terrestrial productivity is largely associated with increased DOC export. In Stordalen catchment we tested this hypothesis with terrestrial productivity approximated by the NDVI. The hypothesis holds some truth, however; it (NDVI) is not a strong indicator of DOC export on its own. Using simple regression methods this becomes clear. Stringent statistical tests using linear mixed regression modeling support the use of land cover as a primary indicator of DOC export in a catchment. Variables such as NDVI, elevation, and slope should be used in conjunction with land cover data (e.g. mires, forests, lakes). In particular, the role and influence of mires is highlighted. In this study we found that when terrestrial productivity increases, the DOC concentration in recipient waters also slightly increases. However, if peatlands are affected by increased precipitation, altered hydrology, and frost conditions etc., that might have a larger bearing on the lateral transfer of DOC. However, more research is needed in other northern catchments to reach a conceptual understanding of the controls influencing DOC release which are strongly affected by climate change. This does not have to be restricted to northern catchments but research from other geographic areas with differing spatio-temporal configurations can enrich the narrative of the terrestrial lateral transfer of carbon.

## Supporting information

S1 Text Source data citation aprroval for the vegetation map of Stordalen catchment, Sweden. (PDF)

S2 Text ORNL DAAC graphics citation communication. (PDF)

S1 Protocol Guide to attribution of geodata by Lantmäteriet. (PDF)
