# Supplementary material for: Dissolved organic carbon in streams within a subarctic catchment analysed using a GIS/remote sensing approach
Source: PLoS One. 2018 Jul 6;13(7):e0199608. doi: 10.1371/journal.pone.0199608 (PMC6034823; doi:10.1371/journal.pone.0199608)
Supplement: S1 Text — (PDF) [file pone.0199608.s001.pdf]

Pearl MZOBE

From: Jing Tang <lu.gistangjing@gmail.com>
Sent: Monday, March 05, 2018 12:13 PM
To: Pearl MZOBE
Subject: Re: Vegetation Classification

Hi Pearl,

thanks for your good wishes. we are doing fine. I hope you also!
The vegetation map was made by one of Andreas master student. Andreas sent me this file, so he is on my coauthor in Tang et al., 2015.
I think it is right to cite the Tang etal. 2015 paper.
Hope everything goes smoothly with you!

Cheers,
Jing Tang

bio-email: jing.tang@bio.ku.dk
Tel: +45 35334606

Terrestrial Ecology Section
Department of Biology
University of Copenhagen
Universitetsparken 15
DK-2100 Copenhagen Ø, Denmark

Centre for Permafrost (CENPERM)

Øster Voldgade 10

DK-1350 Copenhagen K, Denmark

On Mon, Mar 5, 2018 at 11:43 AM, Pearl MZOBE <pearl.mzobe@nateko.lu.se> wrote:

Hi Jing

Hope all is well and that Lupin is growing and keeping you guys on your toes.

I have a quick question regarding the vegetation classification shapefile that I got from you a while back (shown below). Do I cite you or is there another citation for it? I am sorting out copyright issues for a publication. I currently have it as Tang et al. (2015).

(a)

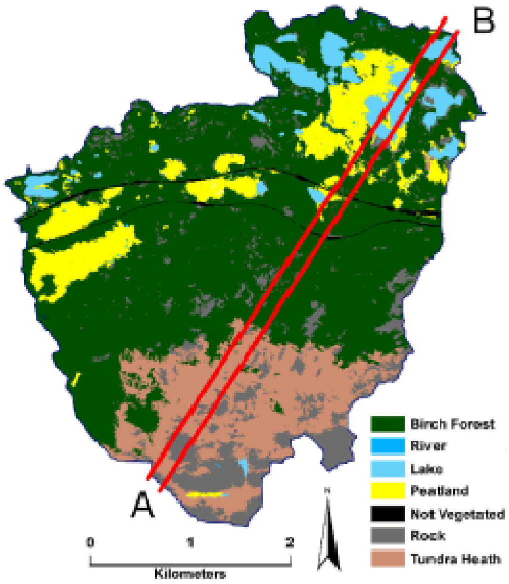

Any direction that you can provide is greatly appreciated. Thanks.

Warm Regards,

Pearl Mzobe.
