# Supplementary material for: Dissolved organic carbon in streams within a subarctic catchment analysed using a GIS/remote sensing approach
Source: PLoS One. 2018 Jul 6;13(7):e0199608. doi: 10.1371/journal.pone.0199608 (PMC6034823; doi:10.1371/journal.pone.0199608)
Supplement: S2 Text — (PDF) [file pone.0199608.s002.pdf]

Pearl MZOBE

From: Jim Lay <support-ornl@earthdata.nasa.gov>
Sent: Friday, March 02, 2018 3:26 PM
To: Pearl MZOBE
Cc: uso@daac.ornl.gov
Subject: [#81593]: Citing graphics from MODIS Subsets

Hello Pearl.
Thank you for your interest in the ORNL DAAC!
The citation you referenced is sufficient for both the data and the graphic. For the graphic, you can also note that it was modified from the ORNL DAAC Global Subsetting Tool", if you would like.
Thank you again!

Sincerely,
Jim Lay
Environmental Informatics Analyst,
Oak Ridge National Laboratory
layjd@ornl.gov
https://daac.ornl.gov

Ticket History **Pearl MZOBE** (Client) Posted On: 02 March 2018 04:05 AM

Hello

I have modified a graphic that was available when I downloaded a subset from the Global Subsets Tool online. It is the "R_MODIS_Plots_250m_16_days_NDVI_2.jpeg.jpg."

Do you have a citation policy for graphics?

I currently have the citation for the data as:
ORNL DAAC. 2008. MODIS Collection 5 Land Products Global Subsetting and Visualization Tool. ORNL DAAC, Oak Ridge, Tennessee, USA. Accessed April 13, 2017. Subset obtained for MOD13Q1 product at 68.3554N,19.047E, time period: 2000-02-18 to 2017-03-06, and subset size: 6.25 x 6.25 km.
http://dx.doi.org/10.3334/ORNLDAAC/1241

I am trying to have this published and want to ensure that there are no copyright problems. Any direction you can provide is greatly appreciated.

Sincerely,

Pearl Mzobe.

P.S. I have attached the original and modified graphic.

Ticket Details

Ticket ID: 81593
Department: ORNL DAAC
Type: General
Status: Open
Priority: Normal

Helpdesk: https://support.earthdata.nasa.gov/index.php?
