# Supplementary material for: Dissolved organic carbon in streams within a subarctic catchment analysed using a GIS/remote sensing approach
Source: PLoS One. 2018 Jul 6;13(7):e0199608. doi: 10.1371/journal.pone.0199608 (PMC6034823; doi:10.1371/journal.pone.0199608)
Supplement: S1 Protocol — (PDF) [file pone.0199608.s003.pdf]

Geodata
SVERIGE BIT FÖR BIT

LANTMÄTERIET

Guide to attribution of geodata, version 1.0
Date: 22/04/2016

Source attribution and copyright for digital maps and geodata

The recommendations in this document have been drawn up by Lantmäteriet, the Swedish Mapping Cadastre and land Registration Authority, in consultation with the Association of Swedish Higher Education (SUHF) geodata working group, the Swedish University of Agricultural Sciences (SLU) and the Earth Sciences Library at Uppsala University.

The GET e-service – a digital library for geodata

The Swedish University of Agricultural Sciences (SLU) provides an e-service, the 'Geodata Extraction Tool' or GET, to download maps, aerial photographs (orthophotos) and data from various authorities, including geological data, nautical charts and population data. The word geodata is often used as a collective term. The GET service may be compared to a library, with the maps, images and geodata that can be downloaded from the service corresponding to the books on the shelves. Information on the geodata that is included (metadata) can be found on the Geodata portal, and represents the library catalogue. There is also a link to GET.

Source attribution

When you download geodata from the GET service and use it as background images, or when parts of datasets are included in a new image published in papers, reports or articles, you must always state who the geodata comes from (source attribution). You do this by citing the authority which produces and provides the information. Source attribution is intended to provide the reader with details of who created the data, and in many cases, when it was created. If you write articles for scientific and other journals, you should use the journal's template for quoting references. These templates may differ widely from each other.

So SLU or GET should not be given as the source when you retrieve data via the GET service, and you should not describe how the data was obtained. Geodata can be supplied in many different ways, of which downloading via the GET service is one and downloading via the Geodata portal and other public portals are others.

Copyright

If copyright is claimed by the party providing the data, you must also indicate this by means of the © symbol and the name of the authority concerned, e.g. *© Lantmäteriet*. The copyright mark is intended to alert the reader to the fact that the data is protected by copyright. This means that further use and dissemination of the data to third parties generally requires consent from or an agreement with the party claiming copyright.

Lantmäteriet, the Swedish Maritime Administration, the Geological Survey of Sweden (SGU) and Statistics Sweden (SCB) claim copyright. When you publish or otherwise distribute data under the conditions of use from these authorities, you must show © *Authority*. This also applies to data from many other authorities and organisations, and it may also apply to data provided free of charge or as 'open data'. This will be stated in the licence conditions for the dataset in question. You will find

these terms and conditions in the metadata for the dataset (or icon when you search within the Geodata portal) under *Restrictions.* This might say e.g. '*Access restrictions*: Copyright; *Restrictions on use*: Creative Commons - Attribution (CC BY, version 4.0)'. If you write © *Authority,* this is an approved acknowledgement of copyright.
Note that some data that you retrieve via GET is open data and can be downloaded via the data producer's website. That means that it may be used under conditions as set out in the Creative Commons - Attribution licence (CC BY), for example. The licence means that, as long as you quote the source, you can use, distribute, convert, modify and add to the producer's open data, even in a commercial context. Data not classed as open cannot be distributed in the same way; see full terms and conditions in the licence agreement for research, education and cultural use or the abbreviated terms that you agree to when you order from the GET service. So you need to know what conditions apply to the data you are using (held in the metadata for the product).

Name of data source and map product

To indicate correct sources for the mapping information or geodata used, the correct name of the data source or product should be quoted, e.g. *GSD height data, grid 2+*. The product names for all data freely available to universities, colleges and arts organisations are listed in the schedule of products and services (see page 2-6 in the appendix). The product name also appears in the product description (or equivalent) provided in the 'Docs' folder together with the data from the GET service. The 'ReadMe' text file accompanying every delivery contains a link address for more information on the products from the data producer; in some cases, product descriptions are also available in English. Note that the year and month in the folder for ordered data refer to when the data was supplied to SLU and do not always reflect the creation/publication year.

You should include this information along with the image in your paper/report/article.

Geodata is often obtained from many different sources and processed into a composite map image. Copyright applies to the constituent datasets, but in a new composite map image it may be hard to see what data comes from which source. You should cite all sources in the caption to the image.

Along with the map image, in practice generally in the caption, you should state (in the following order)**:**

- Data source(s), e.g. 'GSD overview map', 'Soil types 1:25,000 - 1:100,000'
- Copyright where applicable, by showing the © (copyright) mark
- The authority the data comes from, e.g. Lantmäteriet, SCB or SGU.

The reference number of the licence agreement no longer has to be quoted.

Remember that a key to the symbols used and a scale should be included with the map image where possible.

Instructions on how captions should be written may vary, but a general rule is that the text should be detailed enough for the image and its origin to be understood without needed to look elsewhere in the document. So it makes sense to show the product name and the scale or resolution for the data sources used. It is usually helpful to give a year too, to indicate how up-to-date the map is.

These instructions also apply if you publish a background image, such as an aerial photo, an overview map, contour data or a thematic map together with your own data in the same figure.

If you have many figures, you can include the attribution in an index of figures
If you have several similar map images from the same source(s), you can include a collective source attribution in an index of figures or illustrations, either after the table of contents at the start of the document or as part of the list of references at the end.

Source attribution is illustrated below with some slightly modified examples from a paper from Uppsala University.

Example 1. Background image from Lantmäteriet together with own data:
You have used contour data from Lantmäteriet to create new data, in this case older coastlines, and are illustrating this with an aerial photo from Lantmäteriet as background.

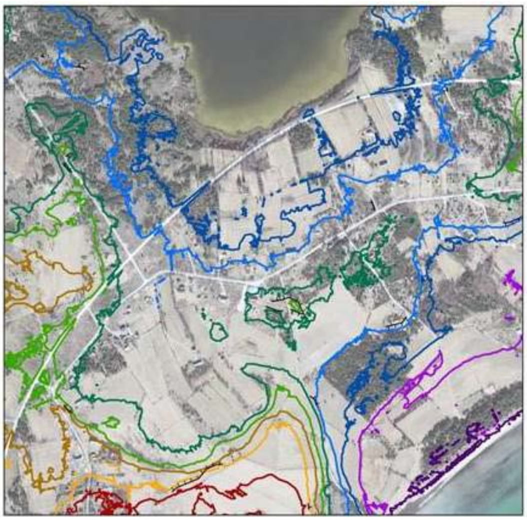

Figure 1. Land uplift map with cliffs and coastlines at 500-year intervals back to 4,500 years ago, created from GSD height data, grid 2+ © Lantmäteriet. Background image: GSD orthophoto, 1m colour © Lantmäteriet (2014).

Comments: For image and contour data, the geometric resolution (pixel size) should be stated; no scale is required for these sources. The year when the data was collected (scanned, photographed) should also be included if it can be derived from the data producer's metadata.

Example 2. Map information from one or more different sources in a new composite map
You have used ‘concentrations of holiday homes’ from 2010 (Statistics Sweden) and wells (SGU) and are publishing an image in which data from these sources has been combined and displayed with the topography from the GSD property map (Lantmäteriet) as background.

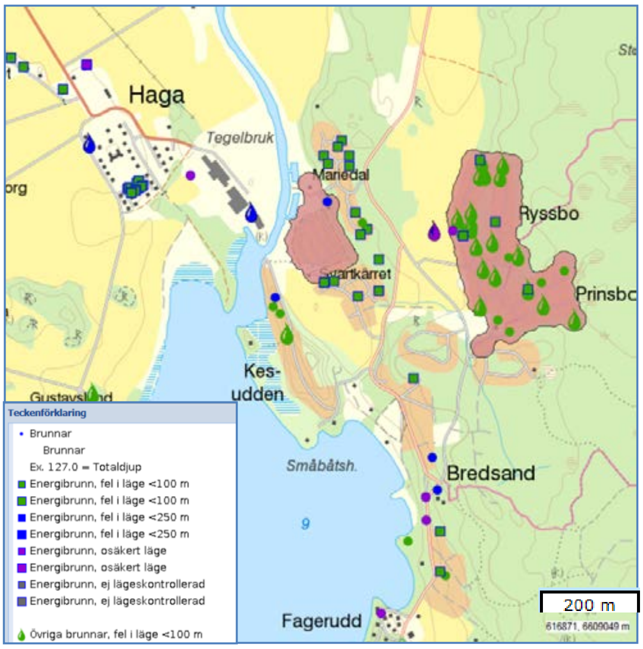

Figure 2. Wells close to concentrations of holiday homes (in pink on the map).
Wells © Geological Survey of Sweden; Concentrations of holiday homes © Statistics Sweden (2010); Background map: GSD topographical property map, scale interval 1:5,000 - 1:20,000 © Lantmäteriet (2015).

Information on geodata (metadata)

Remember also that information on the geodata (metadata) used is available from the authorities that provide data. You can also find metadata on the Geodata portal. If you need to describe your data source in more detail in the running text, this information may be very useful to the reader. The GET service contains links to many of the sources of metadata on the Geodata portal.

Example 3. Screenshot with map image

If a screenshot is used in a paper or report, it must have a source attribution if a map is shown. In the example below, Lantmäteriet's topographical online map (display service) forms the background map on the Geodata portal. In a display service, the age of the source data displayed may vary, so it may be difficult to quote a year for the latest publication or revision.

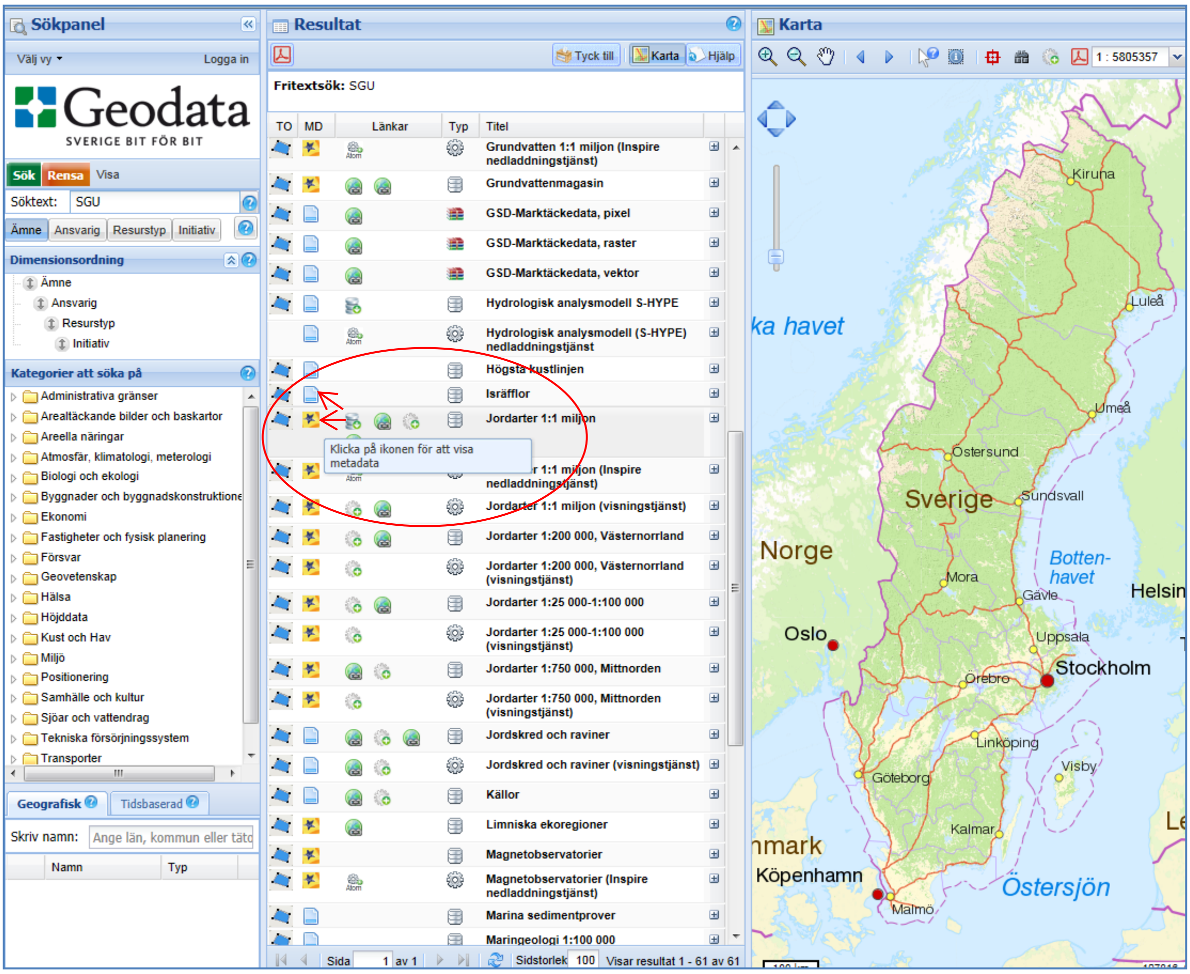

Figure 3. On the Geodata portal you can find metadata information on a given dataset or map by clicking on the icon in the MD (metadata) column. Topographical online map Display © Lantmäteriet.

If you take a screenshot as in the picture above, you must also state this in the *reference list (index of sources*) by indicating the copyright-holder, the date when the page was last updated (where relevant), the page title (in *italics*), the URL or search path, and the date when you accessed the page.

Example:
Figure 3. Lantmäteriet. *Geodata portal*, URL: https://www.geodata.se/geodataexplorer/, [accessed 10/01/2015].
